# De novo *KCNA6* variants with attenuated K_V_ 1.6 channel deactivation in patients with epilepsy

**DOI:** 10.1111/epi.17455

**Published:** 2022-12-05

**Authors:** Vincenzo Salpietro, Valentina Galassi Deforie, Stephanie Efthymiou, Emer O'Connor, Anna Marcé‐Grau, Reza Maroofian, Pasquale Striano, Federico Zara, Michelle M. Morrow, SYNAPS Study Group, Adi Reich, Amy Blevins, Júlia Sala‐Coromina, Andrea Accogli, Sara Fortuna, Marie Alesandrini, P. Y. Billie Au, Nilika Shah Singhal, Benjamin Cogne, Bertrand Isidor, Michael G. Hanna, Alfons Macaya, Dimitri M. Kullmann, Henry Houlden, Roope Männikkö

**Affiliations:** ^1^ Department of Neuromuscular Disease UCL Institute of Neurology, University College London London UK; ^2^ Department of Biotechnological and Applied Clinical Sciences (DISCAB) University of L'Aquila L'Aquila Italy; ^3^ Department of Paediatric Neurology, University Hospital Vall d'Hebron Universitat Autònoma de Barcelona Barcelona Spain; ^4^ Department of Neurosciences, Rehabilitation, Ophthalmology, Genetics, Maternal and Child Health (DiNOGMI) University of Genoa 16124 Genoa Italy; ^5^ Unit of Pediatric Neurology IRCCS, Istituto “Giannina Gaslini” Genoa 16123 Italy; ^6^ Medical Genetics Unit IRCCS, Istituto “Giannina Gaslini” Genoa 16123 Italy; ^7^ GeneDx Maryland Gaithersburg USA; ^8^ Istituto Italiano di Tecnologia (IIT) Genoa Italy; ^9^ Neuropediatrics Unit Centre Hospitalier Universitaire Nantes Nantes France; ^10^ Department of Medical Genetics, Alberta Children's Hospital Research Institute, Cumming School of Medicine University of Calgary Alberta Calgary Canada; ^11^ Departments of Neurology and Pediatrics, UCSF Benioff Children's Hospital University of California California San Francisco USA; ^12^ Centre Hospitalier Universitaire Nantes Service de Génétique Médicale Nantes France; ^13^ Université de Nantes, CNRS, INSERM L'Institut du Thorax Nantes France; ^14^ Queen Square Centre for Neuromuscular Diseases National Hospital for Neurology and Neurosurgery London UK; ^15^ Department of Clinical and Experimental Epilepsy UCL Institute of Neurology, University College London London UK

**Keywords:** K_V_1 Shaker channel family, neurodevelopmental disorder, voltage‐gated potassium channels, whole exome sequencing

## Abstract

**Objective:**

Mutations in the genes encoding neuronal ion channels are a common cause of Mendelian neurological diseases. We sought to identify novel de novo sequence variants in cases with early infantile epileptic phenotypes and neurodevelopmental anomalies.

**Methods:**

Following clinical diagnosis, we performed whole exome sequencing of the index cases and their parents. Identified channel variants were expressed in *Xenopus* oocytes and their functional properties assessed using two‐electrode voltage clamp.

**Results:**

We identified novel de novo variants in *KCNA6* in four unrelated individuals variably affected with neurodevelopmental disorders and seizures with onset in the first year of life. Three of the four identified mutations affect the pore‐lining S6 α‐helix of K_V_1.6. A prominent finding of functional characterization in *Xenopus* oocytes was that the channel variants showed only minor effects on channel activation but slowed channel closure and shifted the voltage dependence of deactivation in a hyperpolarizing direction. Channels with a mutation affecting the S6 helix display dominant effects on channel deactivation when co‐expressed with wild‐type K_V_1.6 or K_V_1.1 subunits.

**Significance:**

This is the first report of de novo nonsynonymous variants in *KCNA6* associated with neurological or any clinical features. Channel variants showed a consistent effect on channel deactivation, slowing the rate of channel closure following normal activation. This specific gain‐of‐function feature is likely to underlie the neurological phenotype in our patients. Our data highlight *KCNA6* as a novel channelopathy gene associated with early infantile epileptic phenotypes and neurodevelopmental anomalies.

**Key Points**

- Trio whole exome sequencing identified de novo variants in *KCNA6* in patients with different degrees of epilepsy and intellectual disability.
- *KCNA6* encodes voltage‐gated potassium channel K_V_1.6 expressed in the central nervous system (CNS) but not associated previously with disease.
- Identified *KCNA6* variants are absent in the control population and predicted damaging with three of four variants affecting pore lining S6 helix.
- Functional analysis showed consistent, dominant defects in channel deactivation, whereas activation was little affected.
- Our data indicate *KCNA6* as a novel gene associated with epilepsy with neurodevelopmental disorder.

## 1 INTRODUCTION

Epilepsy is a neurological disorder that affects ~0.5% to 1.0% of all children up to the age of 16 years of age.^1^ Different behavioral, cognitive, and psychiatric comorbidities are associated with pediatric and adult epilepsy, and in some cases with intellectual disability (ID).^2^ At least 40% of epilepsies are believed to be caused by genetic factors,^3^ of which, only 1% are Mendelian epilepsies.^4^ In the majority of these cases the heritability is still unexplained.^5^, ^6^ Roughly a third of the known genes associated with Mendelian epilepsies encode ion channels.^7^

The voltage‐gated potassium channel (K_V_) superfamily includes more than 40 individual subunits grouped into 12 distinct sub‐families (K_V_1‐12). K_V_ channels conduct the key repolarizing current of action potentials that regulate various physiological processes including neuronal and muscle excitability and the secretion of hormones and neurotransmitters.^8^, ^9^ They consist of four α‐subunits, each of which is composed of six transmembrane helices (S1‐S6).^8^, ^9^ Helices S1‐S4 of each subunit form a regulatory voltage sensing domain (VSD), whereas helices S5‐S6 of all four subunits form a central potassium conducting pore. Upon depolarization, the VSDs undergo conformational transitions that result in channel opening (activation), whereas hyperpolarization closes (deactivates) the pore.^9^ Upon prolonged depolarization, some K_V_ channels undergo inactivation typically mediated by channel N‐terminus occluding the pore (N‐type inactivation). K_V_β‐subunits can modulate α‐subunit activity and induce N‐type inactivation with their N‐terminus.^10^ Pathogenic variants in genes encoding K_V_ subunits may exert one or more effects on the assembly, trafficking, or the kinetics or the voltage dependence of the opening‐closing‐inactivating transitions of the channel, thereby disturbing membrane excitability or action potentials, leading to neurological diseases.^11^, ^12^

The K_V_1 (*KCNA*) subfamily comprises eight homologous subunits (K_V_1.1‐K_V_1.8) that can assemble as homo‐ or heterotetrameric channels with diverse biophysical properties.^13^ K_V_1 channels are delayed rectifiers, as they allow a sustained potassium efflux after membrane depolarization that repolarizes the membrane following an action potential. So far, genetic defects in only K_V_1.1, K_V_1.2, and K_V_1.4 subunits have been identified as established causes of Mendelian neurological diseases.^14^, ^15^ Heterozygous variants in *KCNA1* (K_V_1.1; MIM 176260) have been linked to episodic ataxia type 1 (EA1, MIM 160120), sometimes associated with myokymia or (less commonly) seizures.^16^, ^17^, ^18^ Heterozygous de novo variants in *KCNA2* (K_V_1.2; MIM 176262) were identified as a cause of early‐onset developmental epileptic encephalopathy (DEE 32, MIM 616366).^19^ Both *KCNA1*‐ and *KCNA*2‐related genotypes and phenotypes continue to expand and distinct clinical presentations may arise by the effect of specific mutations on channel expression or function.^15^, ^20^, ^21^, ^22^

*KCNA6* (MIM: 176257) encodes the K_V_1.6 channel predominantly expressed in the central and peripheral nervous system.^23^, ^24^ Unlike the expression of *KCNA1* and *KCNA2, KCNA6* expression is low in putamen and cerebellum (Figure S1), but the expression of these genes coincides in the frontal and occipital cortex where *KCNA6* expression is highest. Expression of K_V_1.6 is increased in a model of epilepsy and has an important impact on network activity.^25^ However, there is no established implication of *KCNA6* in human disease phenotypes. K_V_1.6 channels contain an N‐type inactivation prevention (NIP) domain in the N‐terminus and are insensitive to N‐type inactivation induced by K_V_1.4 subunits or K_V_β1 subunits^26^, ^27^ but sensitive to N‐type inactivation by K_V_β3 subunits.^28^ Herein we describe four unrelated individuals with seizures and/or variable abnormalities of neurological development, in whom exome sequencing detected heterozygous de novo nonsynonymous variants in *KCNA6*.

## 2 MATERIALS AND METHODS

### 2.1 Consent and diagnosis

Affected children were recruited for genetic analysis using whole exome sequencing (WES) at four distinct centers. Written informed consent for genetic sequencing and inclusion in the study was obtained by the parents or legal guardians of all individuals. This study has been approved by the ethics committee of the University College London (07/Q0512/26) and the ethical committees at the other participating centers in Barcelona, Calgary, San Francisco, and Nantes. Clinical, brain imaging, and electroencephalogram (EEG) data were reviewed, and the individuals diagnosed with neurodevelopmental impairment including intellectual disability (ID), developmental delay (DD), and developmental epileptic encephalopathy (EE) were recruited in the different institutions that were participating in the study (individuals 1–4; see Supporting Information for case reports). Based on the International League Against epilepsy (ILAE) classification, an EE was defined in the patients as refractory seizures and cognitive slowing or regression associated with frequent, ongoing epileptiform activity.^29^ ID was defined based on the presence of significant deficits in conceptual, social, and/or practical skills associated with significant deficits in adaptive behavior.^30^ Autism spectrum disorders were defined based on impaired social communication, repetitive behaviors, and signs of sensory abnormalities. The diagnoses were made by specialist pediatric neurology teams at the respective centers. Routine clinical genetic and metabolic screenings performed during initial workup was negative in each case, which warranted further investigation on a research basis.

### 2.2 Next‐generation sequencing and bioinformatics

Genomic DNA was extracted from the whole blood or saliva after informed consent for DNA analysis was provided by the families. Trio WES (index case and his/her parents) was performed in all individuals. Libraries were prepared from parent and patient DNA, and exomes were captured and sequenced by WES on Illumina sequencers. Raw data were processed and filtered with established pipelines. Exonic and donor and acceptor splicing variants were considered for further analysis. Priority was given to rare variants that were present at <1% frequency in public databases, such as 1000 Genomes Project, NHLBI Exome Variant Server, and the Genome Aggregation Database (GnomAD) and had a genomic evolutionary rate profiling (GERP) score >2. Synonymous variants were not considered. Variants were confirmed by Sanger sequencing. Parents of the affected individuals were recruited for segregation analysis, which was carried out using Sanger sequencing. All variants were determined independently by the participating centers. The contributing research centers were connected with help of web‐based tools such as GeneMatcher.^31^

### 2.3 Homology model

The Chain B of 3LUT (structure for the full‐length K_V_1.2, 2.9 Å) with a 73.55% BLAST identity with K_V_1.6 based on the Swiss Model portal^32^ was used as a template to build *KCNA6*. The model underwent steepest descent minimizations to be stopped either when the maximum force was lower than 1000.0 kJ/mol/nm or when 50 000 minimization steps were performed with 0.005 kJ/mol energy step size, with Verlet cutoff scheme, short‐range electrostatic cutoff, and Van der Waals cutoff of 1.0 nm with AMBER99SB‐ILDN force field first with fixed backbone, and then without constraints as implemented in the Gromacs package v. 2016.1. The model was processed by the Orientations of Proteins in Membranes (OPM) database.^33^ Mutants were built by manually mutating relevant residues with Swiss‐PdbViewer^34^ and reiterating the same procedure as described above.

### 2.4 Molecular biology and *Xenopus laevis* oocytes

Rat *Kcna6* clone^23^ was a gift from A Vassilevski (Russian Academy of Sciences, Moscow). Mutations were introduced using the QuikChange site directed mutagenesis kit (Agilent technologies) and verified by sequencing whole *Kcna6*. Messenger RNA (mRNA) was transcribed in vitro using mMessage mMachine kit (ThermoFisher). *X. laevis* oocytes were isolated according to procedures approved by the UK Animals (Scientific Procedures) Act 1986 and injected with 2.5 ng mutant or wild‐type (WT) *Kcna6* or WT *KCNA1* mRNA^22^ and stored in a modified Barth's solution supplemented with penicillin (50 U/ml), streptomycin (50 μg/mL), and amikacin (100 μg/mL). The heterozygous condition was simulated by co‐injecting WT and mutant mRNAs in a 1:1 ratio (2.5 total mRNA).

### 2.5 Electrophysiology

Two electrode voltage‐clamp recordings were obtained 24–72 h post‐injection with GeneClamp 500B amplifier and Clampex 9.2 software (Molecular devices) at room temperature. Records were filtered at 1 kHz and sampled at 5 kHz. Electrode resistance was 0.1–1 MΩ when filled with KCl 3M. Bath solution composition was (in mM): 110 NaCl, 10 KCl, 1.8 CaCl_2_, 10 HEPES, and pH 7.4. –*P*/4 protocol was used to subtract the small leak currents.

The standard protocol to assess the voltage dependence of activation was composed of depolarizing test‐voltage pulses from holding voltage of −80 mV for 250 ms in 10 mV increments and measuring the current amplitude in response to tail voltage of −30 mV. At the tail voltage, WT and mutant channels activate slowly allowing for establishment of a stable baseline after settling of capacitive transients. Voltage dependence and time course of deactivation were assessed using a protocol where channels were fully activated at +30 mV for 250 ms before applying test‐voltage pulses to more negative voltages in 10 mV decrements. Voltage dependence was assessed by plotting the tail current amplitude measured following the test pulses against voltage. Time course of deactivation was assessed by fitting an exponential curve to the current decay during the test‐voltage step. The voltage dependence of activation and deactivation were also assessed using the same protocols, but the duration of the pre‐pulse and the test pulses was only 20 ms and the activating pre‐pulse was to +60 mV.

To assess the voltage dependence of activation or deactivation, the tail current amplitude was plotted against the test voltage and the current–voltage relationship was fit by a Boltzmann function: *y* = 1/(1 + exp (*V*_1/2_ − *V*)/*V*_Slope_). To assess the rate of the deactivation, the current decay was fitted with single exponential function. The data were analyzed using Clampfit 10.7 (Molecular devices), Origin (OriginLabs), and GraphPad Prism software. Data are presented as means ± standard error of the mean (SEM). The parameters were compared via one‐way analysis of variance (ANOVA) with Tukey's multiple comparisons tests and Welch's ANOVA with Dunnett T3 multiple comparison tests with ranked values for nonparametric data.

## 3 RESULTS

### 3.1 Clinical synopsis

Extended case reports and patient videos are provided in the Supporting Information. Genotypic and phenotypic characteristics of the four individuals are summarized in Table 1.

**TABLE 1:** Phenotypes of individuals carrying *KCNA6* de novo variants

	Individual 1	Individual 2	Individual 3	Individual 4
Country of origin	Canada	Spain	France	USA
Gender	Male	Female	Male	Female
Age at last follow‐up	7 years	22 years	6 years	2 years
Variant	(NM_002235.3):c.783C>G; (p.Asp261Glu)	(NM_002235.3):c.1366G>C; (p.Val456Leu)	(NM_002235.3):c.1346C>T; (p.Thr449Ile)	(NM_002235.3):c.1339G>T; (p.Val447Phe)
Inheritance	De novo	De novo	De novo	De novo
Family history	Migraine	–	–	Older sibling with febrile seizures
Other genetic findings	–	–	–	–
Pregnancy	Normal	Normal	Normal	Normal
Birth	Term (cesarean section)	Term (cesarean section)	Term (natural delivery)	Induced at 41 weeks 4 days (natural delivery)
Neonatal course	Admitted to neonatal care unit due to poor oral intake	Normal	Normal	Normal
Birth weight	2000 g	2900 g	2900 g	3885 g
Birth OFC	NA	NA	35 cm	35 cm
Follow–up OFC	50th	<3rd centile	50th	50th
Other	Short stature <3rd	–	–	–
Developmental delay	Yes	Yes	Yes	No
Sitting (months)	12	10	NA	7
Walking (months)	18	24	17	12
Language	First words at age 3 years	First words aged 2 years	Speech delay	Appropriate. First words by 12 months; 30 words by 15 months
Social communication	Late social smile (6 months)	–	–	Appropriate
Intellectual disability	–	Mild	Mild	No
Seizure types	–	Focal, tonic, tonic–clonic	Focal, absences, tonic–clonic (fever)	Focal (fever)
Onset of seizures	–	3 months	5 months	3 months
Autism spectrum disorder	Yes	No	No	No
Behavior	Repetitive behaviors, selective eating habits	Normal	Normal	Normal
Other	–	Joint hypermobility	Learning difficulties	–
Dysmorphic features	Triangular face, pointed chin, thin upper lip	Mild retrognathia, gingival hyperplasia	No	No
Neurological examination	Motor clumsiness	Tremor	Normal	Normal
MRI	Normal	Normal	Normal	Normal
EEG	Normal	Focal and diffuse epileptic abnormalities	Focal and diffuse epileptic abnormalities	Left posterior epileptic abnormalities
Other	Congenital macrocytic anemia	–	–	–

Abbreviation: EEG, electroencephalogram; MRI, magnetic resonance imaging; OFC, occipital frontal circumference.

In all affected children, pregnancies, family histories and birth histories were unremarkable. Neurodevelopmental impairment had been evident since early infancy in three affected individuals (1–3), who presented delay of gross motor skills such as sitting and crawling and achieved autonomous walk between 18 months (individuals 1 and 3) and 24 months (individual 2) of age. Language impairment was also present in individuals 1–3, who attained meaningful speech production late (between 2 and 3 years of age). Late social smile was noticed during the first months of life in individual 1, who showed impaired social communication, repetitive behaviors, and autistic features. Mild intellectual disability was diagnosed during childhood in individuals 2 and 3.

Seizures occurred in the first months of life (between the third and the fifth month of life) in three children (individuals 2–4) and presented with different semiology (Table 1). Individual 2 first experienced multiple focal seizures per day, each lasting a few seconds and characterized by brief upward gaze deviation, uni‐ or bilateral upper limb flexion, and associated with altered level of consciousness; these episodes started at the age of 3 months and usually occurred upon awakening. During infancy and early childhood, she also manifested drug‐resistant generalized (tonic, tonic–clonic) seizures. Individual 3 had multiple seizures per day, lasting a few seconds, and in most cases fever‐related; these episodes started at the age of 5 months and were characterized by clonic movements of the upper extremities with altered level of consciousness. In addition, she manifested absences from age 9 months and tonic–clonic seizures (triggered by fever) from age 22 months. Individual 4 presented at age 3 months with febrile and afebrile focal seizures with eye deviation and asymmetric tonic extension of the upper extremities, followed by bilateral asynchronous/synchronous clonic movements; these episodes, usually lasting seconds to minutes and occurring from drowsiness/sleep, were characterized on EEG by ictal onset over the left posterior region (Figure S2). Several anti‐seizure drugs, including valproic acid, clobazam, lamotrigine, carbamazepine, and ethosuximide, have been trialed in individuals 2–4 (see Supporting Information case reports); beneficial effects of ethosuximide treatment were noticed in individual 2, who has been seizure‐free since the age of 5 years and has had normal follow‐up EEG findings. In addition, individual 4 responded to carbamazepine.

The clinical features summarized are consistent with a diagnosis of variable epileptic phenotypes and/or neurodevelopmental anomalies in the four affected individuals. Other distinctive clinical features were noted in individual 1, who showed macrocytic anemia and distinctive facial features, including pointed chin, mildly flat midface, thin upper lip, and short stature (Supporting Information). Extensive genetic and biochemical investigations for a range of genetic conditions, including non‐syndromic intellectual disabilities, genetic epilepsies, and neurometabolic disorders, were normal. In addition, brain magnetic resonance imaging (MRI) studies were performed in three individuals (individuals 2–4) and did not disclose any abnormality.

### 3.2 Molecular genetic analysis and protein prediction

**FIGURE 1:**
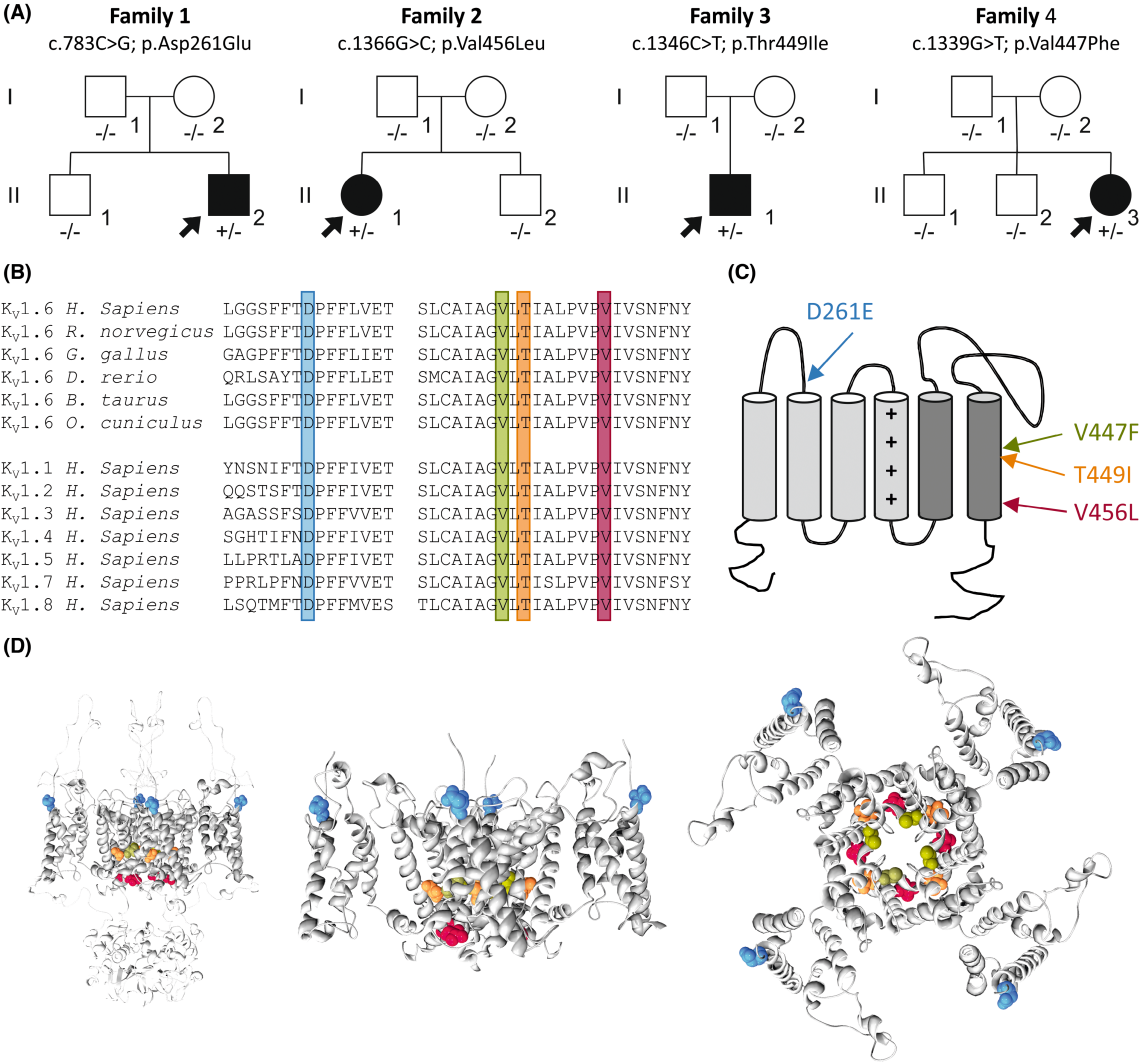
*KCNA6* intragenic de novo variants identified in this study. (A) Family trees on individuals carrying de novo *KCNA6* variants. (B) Multiple alignment showing complete conservation across species and K_V_1 homologues of the residues affected by the variants identified in this study (highlighted). (C) Cartoon topology of the human K_V_1.6 channel indicating the positions of the variants identified in this study. (+) signs indicate positively charged arginine residues in the S4 transmembrane helix. (D) Homology modeling of K_V_1.6 channel showing the full‐length channel in membrane plane (left panel), only the transmembrane domain in membrane plane (middle panel), and from above the membrane plane (right panel). The affected residues are shown in spheres and colored as indicated in C.

Four de novo non‐synonymous variants in *KCNA6* were identified in individuals 1–4, [NM_002235.3: c.783C>G (p.Asp261Glu), c.1366G>C (p.Val456Leu), c.1346C>T (p.Thr449Ile), c.1339G>T (p.Val447Phe), respectively] (Figure 1A, Table S1). The de novo *KCNA6* variant in individual 1 was diagnosed via clinical trio‐based WES through the commercial testing lab, Blueprint Genetics. In individuals 2–4, following their respective analysis pipelines,^35^, ^36^, ^37^ participating centers generated a list of candidate variants filtered against variants from public databases according to modes of inheritance, and then compared their results through international research networks. All the four variants were absent from the GnomAD database, and all displayed high conservation (mean: GERP 5.14) and in silico pathogenic predictors (mean: CADD_Phred 24.14) scores (Table S1). In the Genome Aggregation Database (gnomAD v2.1.1; https://gnomad.broadinstitute.org/) *KCNA6* stands as a constrained gene intolerant to missense variation (*Z*‐score: 3.05). All identified variants affect residues that are highly conserved across different species and K_V_1 subunits (Figure 1B). Three of four de novo non‐synonymous variants (p.Val447Phe, p.Thr449Ile, p.Val456Leu) cluster within the pore lining S6 helix, and one variant (p.Asp261Glu) is located in the S1–S2 loop (Figure 1C,D).

### 3.3 Electrophysiology studies

**FIGURE 2:**
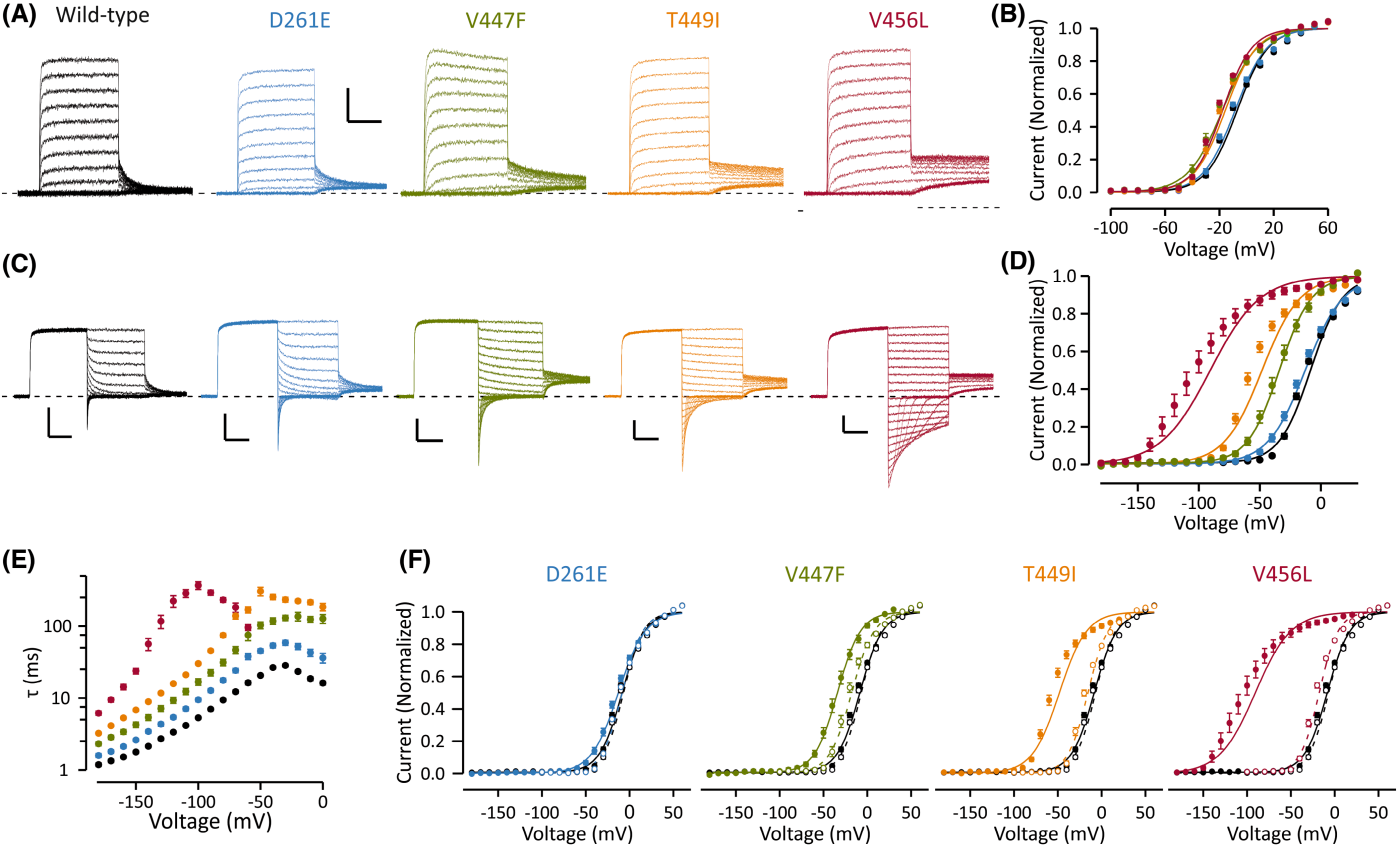
Activation and deactivation properties of wild‐type and mutant K_V_1.6 channels. Data for wild‐type channels are shown in black, for D261E channels in blue, V447F channels in green, T449I in orange, and V456L in red. Data are shown as mean ± SEM. Number of cells is shown in Table 2. (A) Representative traces depicting activation of wild‐type and mutant channels. Voltage protocol consists of 250 ms test voltage steps in 10 mV increments from holding voltage of −80 mV and a tail voltage step to −30 mV. The scale bars are 100 ms (*X*) and 2 μA (*Y*). (B) Voltage dependence of activation was assessed using data from recordings by plotting the current at the beginning of the tail voltage step against the test voltage. (C) Representative traces depicting deactivation of wild‐type and mutant channels. Voltage protocol consists of 250 ms depolarizing pre‐pulse to +30 mV from holding voltage of −80 mV, 250 ms test voltage steps in 10 mV decrements, and a tail voltage step to −30 mV. Scale bars are as in a. (D) Voltage dependence of deactivation was assessed using data from recordings by plotting the current at the beginning of the tail voltage step against the test voltage. (E) Time constant of deactivation is plotted against the test voltage. (F) Voltage dependence of activation (open symbols, data from B) and deactivation (closed symbols, data from D) are plotted for each mutant channel. Note that although for wild‐type and D261E the curves overlap, for other mutant channels the voltage dependence of activation and deactivation is clearly distinct. Solid and dashed lines in B, D, and F show fit of Boltzmann equation to mean data; the dashed lines in F show the fit for activation data and solid lines for deactivation data. Data from individual cells were normalized to the peak amplitude derived from fitting Boltzmann equation to data.

Functional properties of the channel variants were studied in *X. laevis* oocytes using a two‐electrode voltage clamp. The data are summarized in Table 2. The voltage dependence of activation was studied using 250 ms pulses to test voltages and showed an ~−10 mV shift for the variants in the S6 helix compared with WT K_V_1.6 channels (Figure 2A,B). Following the test‐voltage steps, the channel closure at the tail voltage was rapid for WT channels but clearly delayed for the S6 mutant channels (Figure 2A). To study the deactivation, we applied first an activating pre‐pulse for 250 ms to +30 mV and then studied channel closure when stepping to more negative voltages (Figure 2C–E). Compared to WT, the rate of closure of mutant channels was slowed (Figure 2E), and for variants affecting the S6 helix voltage, steps to more negative voltages were needed to close the channel (Figure 2D), demonstrating disrupted channel deactivation. For WT channels, the voltage dependence of activation and deactivation were parallel using 250 ms test pulses. For the channels with mutation in S6 helix, the disrupted deactivation brought forward a hysteresis in voltage dependence of channel gating: voltage dependence of deactivation is shifted tens of millivolts to hyperpolarized voltages compared to voltage dependence of activation following 250 ms voltage steps (Figure 2F). These data suggest that defective deactivation is a specific trait of these K_V_1.6 mutant channels.

**TABLE 2:** Electrophysiology parameters of K_V_1.6 channel variants

Clone	*n*	Activation				Deactivation					
		*V*_1/2_ (mV)	SE	*V*_Slope_ (mV)	SE	*V*_1/2_ (mV)	SE	*V*_Slope_ (mV)	SE	*τ* (@‐80 mV) (ms)	SE
*250 ms pulses*											
WT	18	−8.1	0.7	13.0	0.3	−9.9	0.7	13.6	0.2	9.4	0.4
D261E	16	−9.7	0.8	12.6	0.5	−14.0	1.3	15.3	0.5	17.6***	1.2
V447F	14	−19.8***	1.5	12.3	0.4	−34.4***	2.2	13.0	0.5	31.3***	3.2
T449I	17	−17.8***	0.8	11.4	0.3	−53.6***	1.9	14.9	0.8	74.6***	4.6
V456L	25	−20.2***	0.7	11.1**	0.3	−101.0***	5.1	16.0	1.0	231.6***	16.8
D261E+WT	16	−6.7	1.0	12.8	0.4	−8.2	1.0	13.9	0.5		
V447F+WT	14	−12.6*	0.9	12.5	0.3	−16.2***	0.8	15.7***	0.3		
T449I+WT	4	−10.6	0.9	10.7	1.2	−17.7*	1.4	14.9	1.7		
V456L+WT	10	−14.1	1.7	10.9*	0.6	−19.5**	2.0	16.5	1.1		
*20 ms pulses*											
WT	24	−1.1	0.7	16.3	0.4	−16.1	0.9	24.8	0.5		
D261E	17	−0.7	1.0	15.1	0.6	−32.1***	3.3	26.9	0.8		
V447F	12	−7.2	2.2	13.0***	0.3	−65.3***	3.9	21.8***	0.3		
T449I	16	−9.4***	1.2	12.1***	0.2	−112.5***	3.2	17.3***	0.2		
V456L	14	−8.2***	0.9	12.6***	0.4	−149.8***	10.0	18.5	2.0		
D261E+WT	16	0.4	1.2	16.4	0.3	−13.3	1.8	24.5	0.4		
V447F+WT	16	−5.1	1.0	14.1**	0.3	−39.4***	1.7	26.7	0.4		
T449I+WT	15	−3.9	1.1	13.0***	0.5	−52.2***	4.6	24.1	0.7		
V456L+WT	9	−8.8	3.2	11.5***	0.4	−52.6**	7.7	25.7	0.6		
K_V_1.1	18	−17.9***	0.c9	16.3*	0.4	−23.0***	0.9	18.3	0.3		
K_V_1.1+WT	17	−6.9	0.8	14.8	0.2	−17.9	0.6	19.1	0.3		
K_V_1.1+D261E	18	−13.9***	1.0	15.7*	0.2	−22.0**	0.8	18.3	0.2		
K_V_1.1+V447F	13	−17.5***	0.9	14.7	0.3	−53.7***	1.9	22.8*	0.4		
K_V_1.1+T449I	19	−16.7***	0.9	15.2	0.2	−49.3***	2.3	22.2***	0.7		
K_V_1.1+V456L	12	−20.6***	0.9	14.8	0.3	−54.7***	2.5	26.2***	0.6		

*Note*: Wild‐type K_V_1.6 channel is indicated with WT, co‐expression with K_V_1.6 wild‐type channel is indicated with “+WT”, co‐expression with wild‐type K_V_1.1 channel with “K_V_1.1+”. *p < .05, **p < .01, ***p < .001 when compared to WT data for both 250 ms and 20 ms pulses (excluding K_V_1.1 co‐expression experiments) and compared to K_V_1.1 + WT for experiments involving Kv1.1 channel. Data are presented only for cells where both activation and deactivation protocols were studied.

**FIGURE 3:**
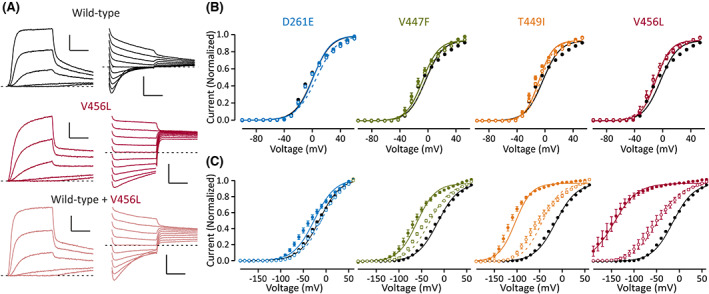
Voltage dependence of activation and deactivation of simulated heterozygous mutant K_V_1.6 channels. Data for wild‐type channels are shown in black, for D261E channels in blue, V447F channels in green, for T449I in orange, and for V456L in red. Data are shown as mean ± SEM. Number of cells is shown in Table 2. Voltage protocols are as in Figure 2A,C, except the duration test pulse and pre‐pulse is 20 ms and the pre‐pulse voltage is +60 mV. (A) Representative traces of oocytes injected with wild‐type (top, black), V456L (middle, red), mRNA alone or both mRNAs together (pink, bottom). Left panel shows current activation in response to test pulses ranging from −40 to 0 mV from holding voltage of −80 mV. Right panel shows current deactivation in response to test pulses ranging from −20 to −110 mV from pre‐pulse voltage of +60 mV. (B) Voltage dependence of activation assessed as in Figure 2A, but the test pulses were only 20 ms in duration. Data are shown for each variant in homomeric (solid symbols and lines) and simulated heterozygous (open symbols, dashed lines) condition. Data for wild‐type channels are shown in black in each graph. (C) Voltage dependence of deactivation assessed as in Figure 2C, but the pre‐ and test pulses were only 20 ms in duration. Symbols and lines are as in B.

To simulate the heterozygous condition of the patients, the oocytes were co‐injected with mutant and WT mRNA at a 1:1 ratio. The simulated heterozygous channels deactivated to almost the same degree as the WT channel during a 250 ms pulse used to study the homomeric channels. We did not attempt to assess the rate of deactivation in simulated heterozygous conditions, as the rate is likely a sum of several rates of K_V_1.6 channel tetramers composed of varying fractions of WT and mutant subunits. When studied with shorter (20 ms) test and pre‐pulses, a significant shift in voltage dependence of deactivation was found for the S6 mutant channels in the simulated heterozygous condition and also for homomeric p.Asp261Glu channels when compared to WT channels (Figure 3). Voltage of half‐maximal activation for the simulated heterozygous channels did not differ from that of WT channels for any mutation (Figure 3B). These data demonstrate that the S6 mutant channels show a dominant effect on channel deactivation and implicate defective deactivation as the main pathogenic mechanism of the simulated heterozygous channels. Using 20 ms steps, the voltage dependence of activation and deactivation differed slightly for WT channels, suggesting that 20 ms is too short for the channel to reach steady‐state at some test voltages. However, the shift between the voltage dependence of activation and deactivation was more pronounced for the simulated heterozygous channels with mutations in the S6 helix (Figure 3).

**FIGURE 4:**
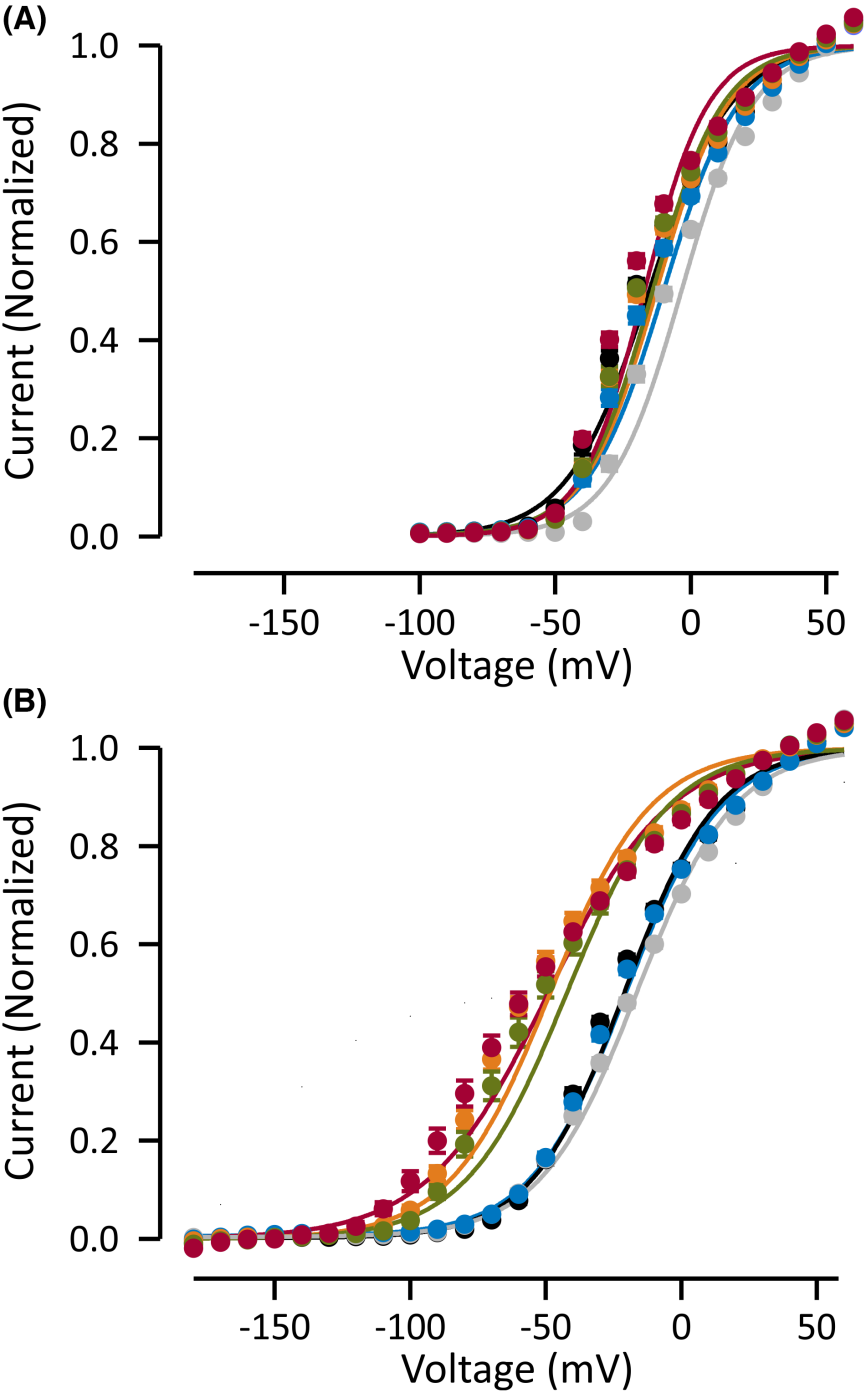
Voltage dependence of activation and deactivation of K_V_1.6 variants co‐expressed with K_V_1.1 subunits. Data for wild‐type K_V_1.1 channels are shown in black, for K_V_1.1 co‐expressed with wild‐type K_V_1.6 in gray, with K_V_1.6‐D261E in blue, with K_V_1.6‐V447F channels in green, with K_V_1.6‐T449I in orange, and with K_V_1.6‐V456L in red. Data are shown as mean ± SEM. Number of cells is shown in Table 2. (A) Shows voltage dependence of activation (voltage protocols are as in Figure 3B) and (B) shows voltage dependence of deactivation (voltage protocols are as in Figure 3C). Solid lines show fit of Boltzmann equation to mean data.

The subunit composition of native K_V_1 channels containing K_V_1.6 subunits is not known, but K_V_1.6 can form functional heteromeric channels with other K_V_1 subunits.^13^, ^27^ We studied whether the defective deactivation features of the mutant Kv1.6 channels are dominant on another K_V_1 isoform by co‐injecting oocytes with both *KCNA6* and *KCNA1* mRNA. We found that all the mutant channels showed a small (~−10 mV) but significant left shift in the voltage dependence of activation compared to when WT K_V_1.6 was expressed together with K_V_1.1 (20 ms pulses) (Figure 4). The voltage dependence of deactivation was left‐shifted more than 30 mV when the K_V_1.1 channel was co‐expressed with S6 mutant K_V_1.6 channels compared to K_V_1.1 channels co‐expressed with WT K_V_1.6 (Figure 4). These data show that the K_V_1.6 mutant channels can exert dominant gain‐of‐function effects on other K_V_1 channels.

Defective deactivation of the mutant subunit is predicted to increase the K^+^ currents upon repetitive stimulation compared to WT channels. Accordingly, although for WT channels the current responses to two 5 ms pulses at 40 Hz were roughly overlapping, for simulated heterozygous S6 mutant channels the current amplitude at holding voltage and in response to the second pulse was clearly augmented compared to the first pulse (Figure S3).

## 4 DISCUSSION

We identified four *KCNA6* variants in patients with varying degrees of neurodevelopmental disorder and seizures. The variability of phenotypes is consistent with that reported in other K_V_‐related genetic channelopathies featuring early‐onset epilepsy variably associated with DD, ID, autism spectrum disorder, and speech difficulties.^15^, ^21^, ^22^, ^38^, ^39^, ^40^ Broad neurological impairment involving multiple developmental domains (motor, cognition, language, behavior) has been described in the context of specific mutations leading to gain‐of‐function effects of different K_V_ channels, including K_V_1.2, K_V_4.2, and K_V_7.3.^19^, ^40^, ^41^, ^42^

The p.Thr449Ile and p.Val456Leu variants, identified in children affected with epilepsy and developmental anomalies (including delayed motor milestones, mild ID, and speech difficulties) exhibited the most pronounced K_V_1.6 gain‐of‐function features among the variants tested in this study. The p.Val447Phe variant, identified in a 2‐year‐old girl affected with epilepsy but without evidence of neurodevelopmental impairment, showed clear gain‐of‐function features but smaller than p.Thr449Ile and p.Val456Leu variants. Lack of neurodevelopmental features may correlate with milder defect on channel function, although the interpretation of the significance of p.Val447Phe on the clinical phenotype may be complicated by the early age of the affected child (individual 4), and long‐term follow‐up will be needed to assess developmental (motor, cognitive, and language) outcomes. Individual 1, found to harbor a de novo non‐synonymous variant in the S1–S2 loop (p.Asp261Glu) did not exhibit seizures and presented neurodevelopmental impairment with delayed motor milestones, speech difficulties, and autistic features. The effects of the p.Asp261Glu variant on channel function were modest compared to the S6 helix mutant channels, although the mutant channel displayed some gain‐of‐function features qualitatively similar to the other mutant channels (Figures 2E and 3C). These defects were no longer detected when the mutant was co‐expressed with K_V_1.6 WT subunits. It is unlikely that p.Asp261Glu underlies the complex clinical phenotype of patient 1 (Table 1, Supporting Information) but it may contribute to the neurodevelopmental features. None of the affected individuals carrying *KCNA6* de novo variants exhibited ataxia or cerebellar involvement, which are frequent features in the neurological and neuroradiological phenotypes related to genetic defects in K_V_1.1^16^, ^17^, ^18^, ^22^ and K_V_1.2 channels.^19^, ^40^ This is consistent with lower cerebellar expression of *KCNA6* when compared to both *KCNA1* and *KCNA2* expression (Figure S1).

Our functional data implicate defective deactivation (gain‐of‐function) as a pathogenic mechanism for *KCNA6*‐related neurological disorders. The defect is dominant, even on another K_V_1 isoform (Figure 4), and is likely to cause specific changes in the firing properties of the neurons carrying the mutant K_V_1.6 channels. Because the relative contribution of K_V_1.6 on total K_V_1 current is not described for any specific neuron, it is difficult to model the effects on firing properties. However, as the mutations minimally affect channel activation, firing threshold and the shape of the first action potential following rest are predicted to be little disturbed. Compromised deactivation will lead to increased K^+^ currents following an initial action potential that likely suppresses the firing of consequent action potentials and/or affects their shape (Figure S3). Other molecular features not assessed in this study, such as N‐type inactivation induced by K_V_β3 subunits,^28^ may be altered by the mutations. In addition, normal inactivation may limit the impact of defective deactivation on cell excitability.

K_V_1.6 channels are broadly expressed in the brain and implicated in a range of neurons and networks (Figure S3).^23^, ^24^, ^25^ Consequently, it is difficult to predict how altered firing of action potentials in neurons expressing K_V_1.6 channel leads to over‐excitability of the brain and which network mechanisms underlie the epileptiform activity. Gain‐of‐function K_V_ channel variants are predicted to dampen the excitability of the neuron. This is predicted to lead to increased electrical activity associated with epilepsy if the GoF variant is expressed in inhibitory interneurons. In this model, dampening of the electrical activity of the inhibitory interneuron results in overactivity of the network controlled by the interneuron. *KCNA6* mRNA and K_V_1.6 expression were increased in the piriform cortex parvalbumin interneurons following induction of epilepsy,^25^ demonstrating K_V_1.6 expression in inhibitory interneurons. Increased expression was associated with reduced diversity of interneuron firing patterns, suggesting that changes in K_V_1.6 currents introduced by mutations may alter the firing patterns of these interneurons and consequently the network activity.

At a molecular level, channel activation is little affected by the mutations, suggesting that the mutations have a minor effect on channel resting state. Following activation, the rate of closure of the mutant channels is slower than for WT channels and voltage dependence of deactivation is shifted to more hyperpolarized voltages compared to the voltage dependence of activation. This hysteretic behavior is compatible with the notion that following channel activation the open state of the channel is stabilized. All three variants affecting the S6 helix (p.Val447Phe, p.Thr449Ile, p.Val456Leu) increase the hydrophobic surface area and the volume of affected residues, potentially stabilizing the open state through increased hydrophobic interactions or steric hindrance in the chains packing. The charge‐conserving p.Asp261Glu variant affects the S1‐S2 loop of the voltage sensor of K_V_1.6 channels, and likely slows the deactivation of the channel by stabilizing the voltage sensor in the activated state.

Gain‐of‐function mutations of ion channels are therapeutically amenable to drugs that block or reduce the current through the overactive channel.^43^ K_V_ channel blockers may thus be of therapeutic interest for individuals affected with *KCNA6*‐related epilepsy with associated gain‐of‐function mechanisms. However, to date and to our knowledge, no K_V_1.6‐ specific small molecule inhibitors of K_V_1.6 have been reported.

## 5 CONCLUSIONS

Our study shows that the de novo *KCNA*6 variants have a specific and similar effect on K_V_1.6‐channel deactivation. This gain‐of‐function effect of the variants identified in the S6 helix effect is dominant, even on another K_V_1 isoform. These data yield solid support for the notion that the K_V_1.6 variants we discovered are pathogenic and causative for a spectrum of infantile‐onset epileptic phenotypes and neurodevelopmental anomalies. Even though our cohort is small, it establishes *KCNA6* as a gene associated with neurodevelopmental disorders and epilepsy. The small number of discovered cases with GoF variants may be a consequence of the specific, unusual effect of the variants on compromising channel deactivation. Low tolerance of *KCNA6* for missense variation suggests that further variants, including loss‐of‐function variants are likely to be discovered associated with neurological disorders.

## AUTHOR CONTRIBUTIONS

Vincenzo Salpietro led the collection of genetic and clinical data. Valentina Galassi Deforie led the collection of electrophysiological data. Stephanie Efthymiou, Emer O'Connor, and Reza Maroofian coordinated the collection and assembly of clinical and genetic data from all participating centers. P. Y. Billie Au collected the clinical and genetic data of individual 1. Anna Marcé‐Grau, Júlia Sala‐Coromina, and Alfons Macaya collected the clinical and genetic data of individual 2. Marie Alesandrini, Benjamin Cogne, and Bertrand Isidor collected the clinical and genetic data of individual 3. Michelle M. Morrow, Adi Reich, Amy Blevins, and Nilika Shah Singhal collected the clinical and genetic data of individual 4. Sara Fortuna created models of K_V_1.6 variants. Pasquale Striano, Federico Zara, and Andrea Accogli contributed to the analysis and revision of the clinical and electroencephalographic data of the SYNAPS Study Group. Michael G. Hanna and Dimitri M. Kullmann oversaw the interpretation of genetic, clinical, and functional data. Henry Houlden and Roope Männikkö oversaw the full study, with Henry Houlden corresponding for the clinical and genetic data and Roope Männikkö for functional data. Vincenzo Salpietro, Valentina Galassi Deforie, and Roope Männikkö wrote the manuscript. All authors read and approved the manuscript. Clinical and genetic data were collected as part of the SYNAPS Study Group.

## FUNDING INFORMATION

This study was supported by the Wellcome Trust (WT093205MA, WT104033AIA) and the Medical Research Council (H.H.). We are also supported by the National Institute for Health Research (NIHR), the University College London Hospitals (UCLH).

## CONFLICT OF INTEREST

Michelle M. Morrow, Adi Reich and Amy Blevins are employees of GeneDX, LLC None of the other author have any disclosures. We confirm that we have read the Journal's position on issues involved in ethical publication and affirm that this report is consistent with those guidelines.

## Supporting information


Data S1

Video S1

Video S2

Video S3

Video S4
