# Supplementary material for: De novo KCNA6 variants with attenuated KV 1.6 channel deactivation in patients with epilepsy
Source: Epilepsia. 2022 Dec 5;64(2):443–55. doi: 10.1111/epi.17455 (PMC10108282; doi:10.1111/epi.17455)
Supplement: Supplementary file 1 — Data S1 [file EPI-64-443-s002.pdf]

Supplemental Data

Subject recruitment and diagnosis

Case reports

Figures

Tables

References

Consortia and network involved in this study

Videos (separate files)

Subject recruitment and diagnosis

The 4 individuals carrying *de novo KCNA6* intragenic variants reported in the present study were recruited from different research groups internationally. Individual 1 was followed up at Alberta Children's Hospital (Calgary, Canada) and genetically investigated at BluePrint Genetics. Individual 2 was followed up at University Hospital "Vall d'Hebron" (Barcelona, Spain) and was genetically investigated by trio- whole exome sequencing (WES) as part of the SYNAPS Study Group (http://neurogenetics.co.uk/synaptopathies-synaps/) initiative. Individual 3 was followed up and genetically investigated at the University Hospital of Nantes (Nantes, France). Individual 4 was followed up at the Benioff University Children's Hospital (San Francisco, USA) and genetically investigated at GeneDx. Routine clinical genetic and metabolic screenings performed during initial workup was negative in each case, which warranted further investigation on a research basis. The study was approved by the ethics committee of the University College London (07/Q0512/26) and additional local ethics committees of the participating centres.

Supplemental Case reports

Individual 1

Patient 1 is a 7-year-old Canadian boy born at term with caesarean section after uneventful pregnancy. He was small for gestational age and required to be admitted to NICU due to poor weight gain. At birth, his occipitofrontal circumference (OFC) was within normal range (50th) and birth weight was 2000 gr (>3rd centile). He has an history of developmental delay since the first months of life. He acquired the ability of sitting autonomously at the age of 12 months and started to walk since the age of 18 months. Abnormal social communication was noticed in the first years of life with late social smiling. Speech delay was present with first meaningful words pronounced at the age of 3 years, but he lately improved with speech therapy. In the first years of life macrocytic anaemia was also noted. He has some distinctive facial features, including pointed chin, mildly flat midface and thin upper lip. At his last follow-up appointment at the age of 8 years he was diagnosed with short stature (3-10th centile); on neurological examination muscle weakness and abnormal motor coordination and clumsiness were noted (e.g., difficulties with running, jumping, etc.) on examination. During his childhood, he displayed some behavioural disturbances and signs of sensory sensitivities and was diagnosed with autism spectrum disorder. There was no history of focal neurological deficits and he never had seizures. He has not showed developmental regression. Trio WES done at BluePrint Genetics did not identify any copy number or single nucleotide variants in disease-causing genes. A *de novo* non-synonymous variant in *KCNA6* [NM_002235.3:c.783C>G; (p.Asp261Glu)] was found in the trios WES data.

Individual 2

Individual 2 is a 22-years-old Spanish female. She was born to healthy, unrelated parents. The rest of her family history is non-contributory. Gestation and term delivery were unremarkable. At birth, her weight was 2900gr (25th centile). Neonatal period was uneventful. At the age 3 of months, she started to present seizures. The episodes occurred on a daily basis and featured brief upward gaze deviation, uni- or bilateral upper limb flexion with altered level of consciousness. The episodes usually lasted a few seconds (less than 10-20 sec) and occurred upon awakening. Between the ages of 6 months and 5 years the patient suffered multiple episodes (up to ten per day) of tonic or tonic-clonic seizures, which were refractory to several drugs, including sodium valproate, vigabatrin and clobazam. Lamotrigine and carbamazepine did not improve her condition. At around 5 years of age, treatment with ethosuximide brought a clear improvement and she has subsequently had good seizure control on this drug. In fact, seizures recurred every time dosing has been reduced in an attempt to taper the medication. Over the years, EEG did show focal or generalized epileptiform abnormalities (not shown), but in recent years have been remarkably normal. Brain CT scan and MRI did not disclose any abnormality. The patient was delayed from early on and she attained independent gait and first meaningful words by age 2. She currently displays mild intellectual disability, is able to read and write simple sentences and attends special school with occupational therapy. She has some distinctive facial features including mild retrognathia and gingival hyperplasia. At her current age, she has poor language syntax and vocabulary and prominent echolalia. She has no major gross or fine motor disabilities, or motor or sensory deficits and she is independent for most basic activities of daily living. She does not show impaired social communication. On neurological examination, she has no motor or sensory deficits, but she displays postural and kinetic high-frequency bimanual tremor, but there is no rigidity, or other extrapyramidal, or pyramidal signs and gait and stance are normal (Supplementary Video 1). She also exhibits some clumsiness, dystonic posturing involving the ankles (left > right) and mild adiadochokinesia (Supplementary Video 1). She underwent multiple metabolic and genetic test including array-CGH which resulted normal. Trio-WES identified a non-synonymous variant in *KCNA6* [NM_002235.3:c.1366G>C; (p.Val456Leu)] that was confirmed *de-novo* by Sanger sequencing.

Individual 3

Individual 3 is a 6-year-old French boy born at term via caesarean section after an uneventful pregnancy. Familial history was negative for neurological or genetic disorders. His growth parameters, including OFC at birth were within normal range. He has a history of mild developmental delay, became able to sit without support at 9 months of age and started to walk autonomously at the age of 17 months. Since the age of 5 months, he presented with multiple focal seizures triggered by fever and characterized by clonic movements at upper limbs lasting few seconds and followed by hypotonia and loss of consciousness. Supplementary Video 2 shows a focal seizure starting with left

eye and head deviation and gaze staring followed by tonic posturing of left side (1’ 10’’) and ending with clonic movements of the upper limbs; the ictal EEG reveals an epileptic activity starting from the right posterior cortical areas and then involving the whole hemisphere. In addition, since the age of 9 months she did also manifest absence seizures (Supplementary Video 2, 1’ 28’’) associated with mild eye rolling and subtle eyelid myoclonia. EEG showed interictal bilateral slow spike and wave discharges. Brain MRI was normal. Valproic acid was started but he continued to experience fever-related convulsive seizures; clobazam and stiripentol add-on did result in good control for 10 months. He was suspected for Dravet syndrome given the history of fever-related epilepsy but *SCN1A* testing was negative. At the age of 22 months, he experienced focal (clonic) seizures recurred and he also experienced tonic-clonic seizures with fever. Increased valproic acid and stiripentol dosages were effective. His language and cognitive skills are impaired, he started to pronounce first words at the age of 3 years and first sentences at the age of 5 years. He currently attends speech therapy sessions. At school, learning difficulties and impaired concentration were noticed and he is supported by a psychomotor therapist. At the last follow up (6 years), the child exhibits clumsiness and difficulties in fine and global motor skills. His metabolic screening and array-CGH were normal. WES identified a *de novo* non-synonymous variant in *KCNA6* [NM_002235.3:c.1346C>T; (p.Thr449Ile)] that was confirmed by Sanger sequencing.

Individual 4

Individual 4 is a 2-year-old American girl born via vaginal birth after induced labor at 41 weeks of gestation. Pregnancy was uncomplicated and at birth her growth parameters were all within normal range. Since the age of 3 months, she developed febrile and afebrile focal seizures with eye deviation, perioral cyanosis and asymmetric tonic extension of the upper extremities followed by bilateral asynchronous/synchronous clonic movements and sometimes associated with facial twitching, perioral clonias and oral automatisms (Supplementary Videos 3 and 4). These episodes, usually lasted seconds to minutes and occurred from drowsiness/sleep. After each episode, the girl is frequently lethargic and drowsy. At the age of 5 months, ictal EEG recorded a focal seizure, starting from the temporal anterior areas (Figure S2A) and rapidly involving the motor cortex (Supplementary Figure 2B); 20 seconds later the seizure involves both hemispheres (Figure S2C) and ends with a diffuse slowing of cortical activity (Figure S2D). Therapy with levetiracetam was ineffective and then switched to carbamazepine with benefit. Her developmental history is so far normal and age-appropriate. At her last follow-up appointment at the age of 2 years, neurological examination was normal. Brain MRI studies and metabolic screening were unrevealing. She underwent trio-WES at Genedx and this led to the identification of a *de-novo* non-synonymous variant in *KCNA6* [NM_002235.3: c.1339G>T; (p.Val447Phe)]. Variant was confirmed by traditional Sanger sequencing.

Supplemental Figures

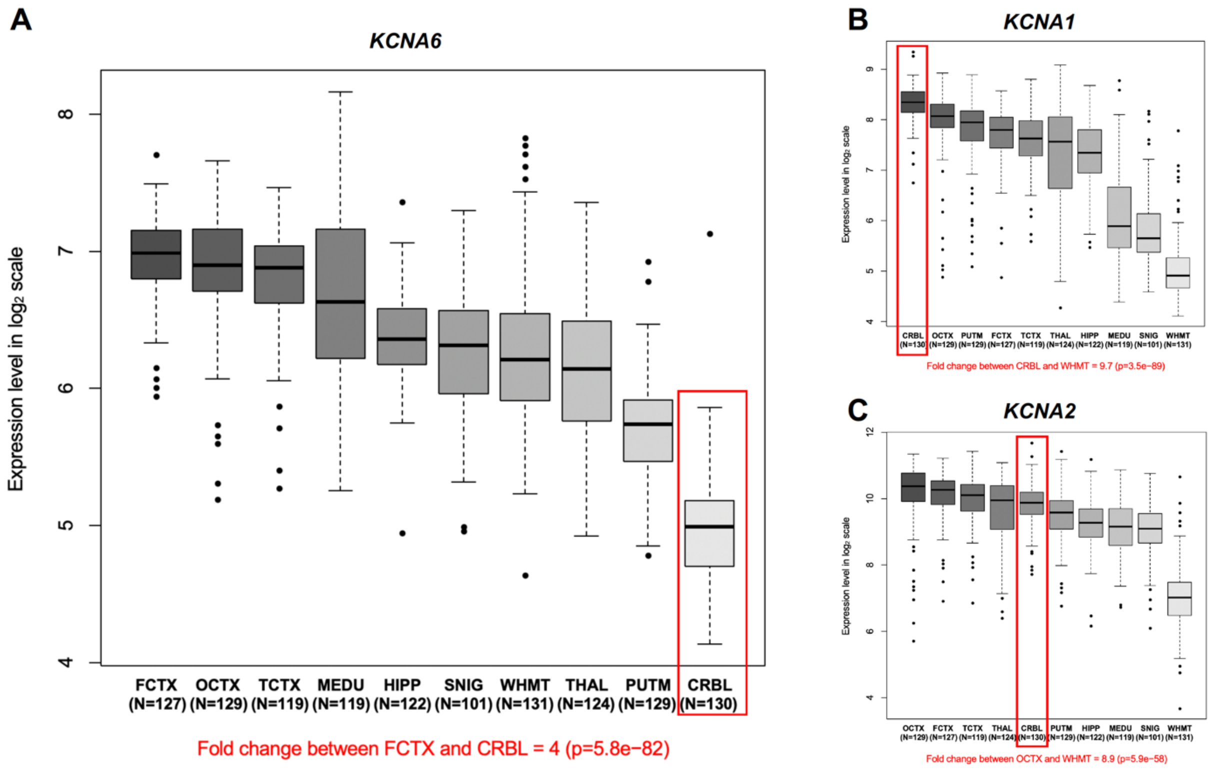

Supplementary Figure 1. Brain expression values of *KCNA6* compared to *KCNA1* and *KCNA2*. To examine *KCNA6* expression across central nervous system (CNS) regions, we used microarray data (Affymetrix Exon 1.0 ST) from human post-mortem brain tissues as previously described[1] and compared to brain expression data of *KCNA1* and *KCNA2*. This analysis showed the highest *KCNA6* expression in the frontal and occipital lobes and lowest expression in putamen and cerebellum. FCTX=frontal cortex, OCTX=occipital cortex, TCTX=temporal cortex, MEDU=medulla, HIPP=hippocampus, SNIG=substantia nigra, WHMT=intralobular white matter, THAL=thalamus, PUTM=putamen, CRBL=cerebellum.

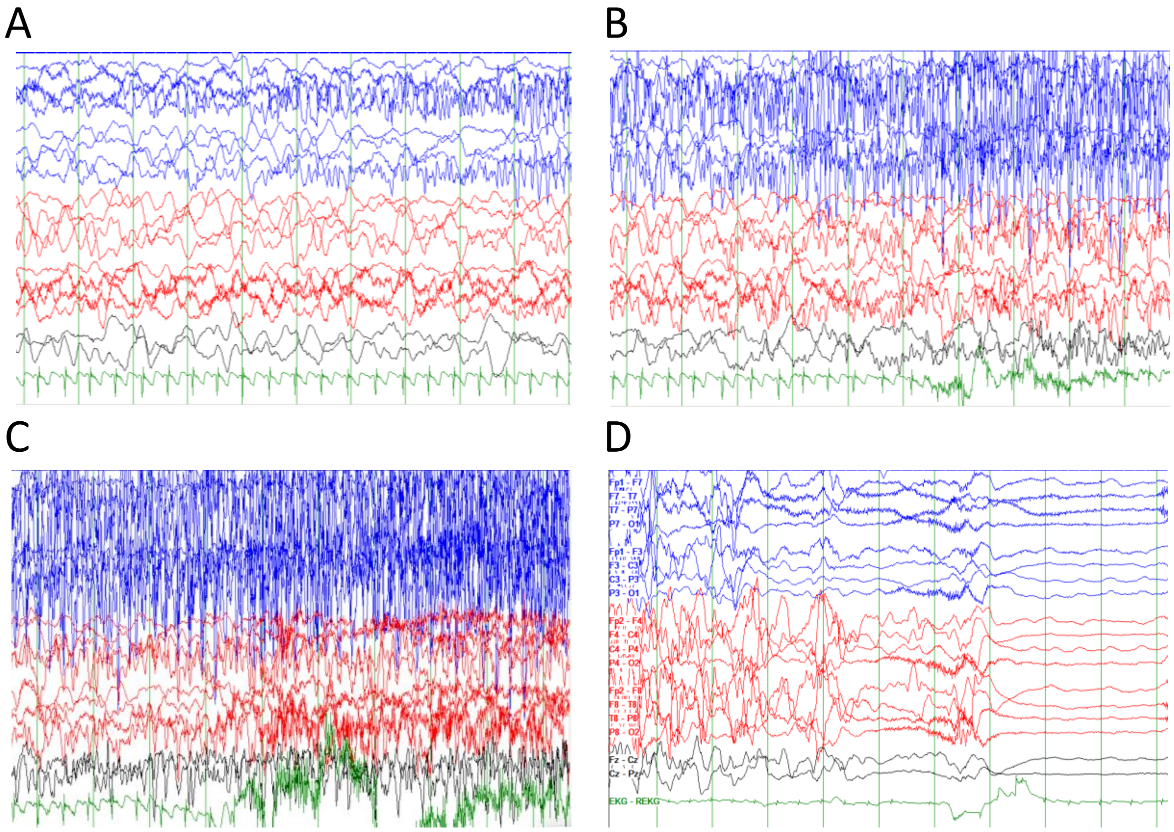

Supplementary Figure 2. Ictal EEG of Individual 4. Ictal EEG record of a focal seizure of individual 4 at the age of 5 months, starting from the temporal anterior areas (A) and rapidly involving the motor cortex (B); 20 seconds later the seizure involves both hemispheres (C) and ends with a diffuse slowing of cortical activity (D).

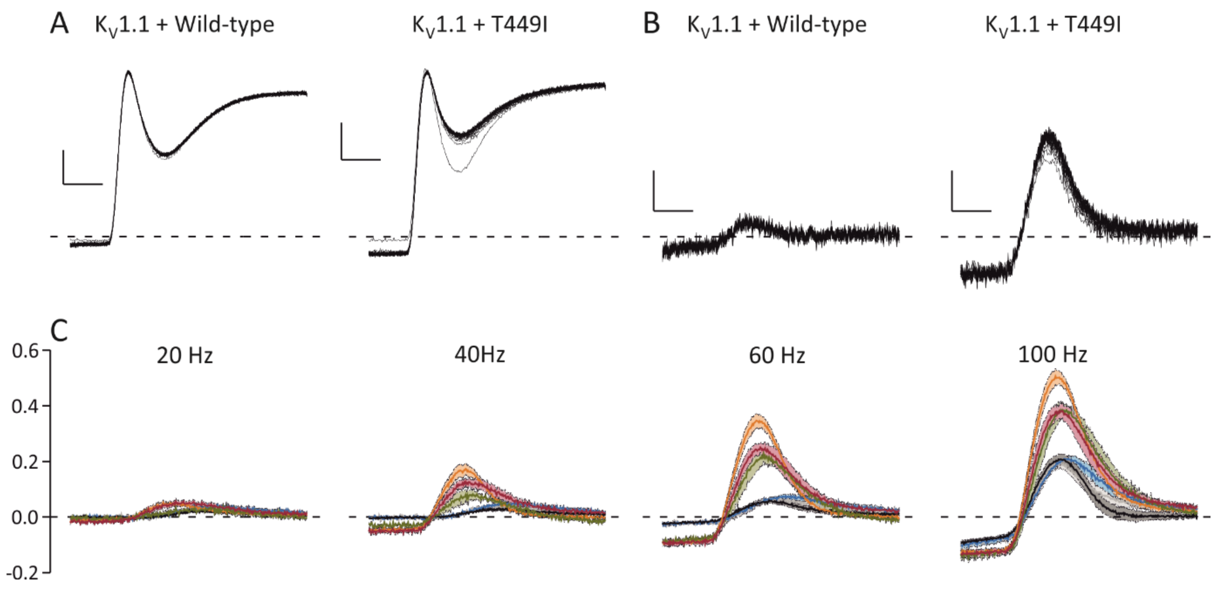

Supplementary Figure 3. Currents responses to repetitive 5 ms pulses (A) Simulated heterozygous channels composed of wild-type K_V1.1 and either wild-type (left) or T449I (right) K_V1.6 channels. Protocol consists of 30 5 ms pulses to +40 mV from holding voltage of -80 mV applied every 15 ms. Capacitive currents at the beginning of the test pulse can be seen as –*P*/4 subtraction protocol was not applied. Note that while the current responses of all the pulses are overlapping for channels containing wild-type K_V1.6 channel, there is a clear increase in current amplitude for K_V1.1+T449I channel following the first pulse. Scale bars: x: 1 ms, y: 5 µA. B) Data from (A) but the current of the first trace is subtracted from the following pulses. Note large increase in K_V1.1+T449I currents before and during the test pulses. Scale bars: x: 1 ms, y: 2 µA. C) Current increase following the first pulse for each K_V1.6 variant co-expressed with K_V1.1 wild-type channel (WT K_V1.6 (grey), D261E (blue), V447F (green), T449I (orange), V456L (red)). K_V1.1 WT is shown in black) at indicated frequencies. For each cell the mean current increase of pulses 2-30 compared to the first pulse is measured and normalised to the peak current amplitude at the end of the voltage pulse to +40 mV. Data is mean ± SEM of 4-9 cells for each K_V1.6 variant and homomeric K_V1.1 channel.

Supplemental Table

Supplementary Table 1. *KCNA6* variants identified in this study

Individual	Genomic coordinates	Transcript and variant (cDNA and protein)	SIFT	PolyPhen	Condel	gnomAD exomes	KCv1.6 Domain	GERP++_NR	CADD_PHRED
1	chr12:4919990-4919990	(NM_002235.3) c.783C>G (p.Asp261Glu)	Tolerated (0.07)	Benign (0.11)	Neutral (0.328)	-	S1-S2 loop	5.12	18.98
2	chr12:4920546-4920546	(NM_002235.3) c.1366G>C (p.Val456Leu)	Deleterious (0)	Probably Damaging (0.997)	Deleterious (0.911)	-	S6 helix	5.18	28.0
3	chr12:4920553-4920553	(NM_002235.3) c.1346C>T (p.Thr449Ile)	Deleterious (0.02)	Probably damaging (0.967)	Deleterious (0.792)	-	S6 helix	5.18	25.3
4	chr12:4920573-4920573	(NM_002235.3) c.1339G>T (p.Val447Phe)	Deleterious (0.01)	Probably damaging (0.992)	Deleterious (0.853)	-	S6 helix	5.09	24.3

Supplemental References

[1] Trabzuni D, Ryten M, Walker R, Smith C, Imran S, Ramasamy A, *et al.* Quality control parameters on a large dataset of regionally dissected human control brains for whole genome expression studies. *J Neurochem*. 2011; 119:275-82.

Consortia and networks involved in this study

The Synaptopathies and Paroxysmal Syndromes (SYNaPS) Study Group

(http://neurogenetics.co.uk/synaptopathies-synaps/)

Study Group Members:

Prof Stanislav Groppa, Department of Neurology and Neurosurgery, Institute of Emergency Medicine, Chisinau, Republic of Moldova.

Dr. Blagovesta Marinova Karashova, Department of Paediatrics, Medical University of Sofia, Sofia 1431, Bulgaria

Dr. Wolfgang Nachbauer, Department of Neurology, Medical University Innsbruck, Anichstrasse 35, Innsbruck 6020, Austria

Prof. Sylvia Boesch, Department of Neurology, Medical University Innsbruck, Anichstrasse 35, Innsbruck 6020, Austria

Dr. Larissa Arning, Department of Human Genetics, Ruhr-University Bochum, Bochum 44801, Germany

Prof. Dagmar Timmann, Braun Neurologische Universitätsklinik Universität Essen, Hufelandstr 55, Essen D-45122, Germany

Prof. Bru Cormand, Department of Genetics, Universitat de Barcelona, Barcelona 08007, Spain

Dr. Belen Pérez-Dueñas, Hospital Sant Joan de Deu, Esplugues de Llobregat 08950, Barcelona, Spain

Dr Gabriella Di Rosa, MD, PhD, Department of Pediatrics, University of Messina, Messina 98123, Italy

Prof. Jatinder S. Goraya, MD, FRCP, Division of Paediatric Neurology, Dayanand Medical College & Hospital, Ludhiana, Punjab 141001, India

Prof. Tipu Sultan, Division of Paediatric Neurology, Children's Hospital of Lahore, Lahore 381-D/2, Pakistan

Prof Jun Mine, Department of Paediatrics, Shimane University, Faculty of Medicine, Izumo, 693-8501, Japan

Prof. Daniela Avdjieva, Department of Paediatrics, Medical University of Sofia, Sofia 1431, Bulgaria

Dr. Hadil Kathom, Department of Pediatrics, Medical University of Sofia, Sofia 1431, Bulgaria

Prof.Dr Radka Tincheva, Head of Department of Clinical Genetics, University Pediatric Hospital, Sofia 1431, Bulgaria

Prof. Selina Banu, Neurosciences Unit, Institute of Child Health and Shishu Shastho Foundation Hospital, Mirpur, Dhaka 1216, Bangladesh

Prof. Mercedes Pineda-Marfa, Servei de Neurologia Pediàtrica, l'Hospital Universitari Vall d'Hebron, Barcelona 08035, Spain

Prof. Pierangelo Veggiotti, Unit of Infantile Neuropsychiatry Fondazione

Istituto Neurologico "C. Mondino" IRCCS, Via Mondino 2, Pavia 27100, Italy

Prof. Michel D. Ferrari, Leiden University Medical Center, Albinusdreef 2, Leiden 2333, Netherlands

Prof. Alberto Verrotti, University of L'Aquila, L'Aquila, Italy

Prof Giangluigi Marseglia, Department of Pediatrics, University of Pavia, IRCCS Policlinico "San Matteo", Pavia 27100, Italy

Dr. Salvatore Savasta, Division of Pediatric Neurology, Department of Pediatrics, University of Pavia, IRCCS Policlinico "San Matteo", Pavia 27100, Italy

Dr. Mayte García-Silva, Hospital Universitario 12 de Octubre, Madrid 28041, Spain

Dr. Alfons Macaya Ruiz, University Hospital Vall d'Hebron, Barcelona 08035, Spain

Prof. Barbara Garavaglia, IRCCS Foundation, Neurological Institute "Carlo Besta", Molecular Neurogenetics, 20126 Milan, Italy

Dr. Eugenia Borgione, Laboratorio di Neuropatologia Clinica, U.O.S. Malattie, Neuromuscolari Associazione OASI Maria SS. ONLUS – IRCCS, Via Conte Ruggero 73, 94018 Troina, Italy

Dr. Simona Portaro, IRCCS Centro Neurolesi "Bonino Pulejo", SS113, c.da Casazza, 98124 Messina, Italy

Dr. Benigno Monteagudo Sanchez, Hospital Arquitecto Marcide, Avenida de la Residencia S/N, Ferrol (A Coruña), 15401 Spain

Dr. Richard Boles, Courtagen Life Sciences, 12 Gill Street Suite 3700, Woburn, MA 01801 USA

Prof. Savvas Papacostas, Neurology Clinic B, The Cyprus Institute of Neurology and Genetics, 6 International Airport Road, 1683 Nicosia, Cyprus

Dr. Michail Vikelis, Iatreio Kefalalgias Glyfadas, 8 Lazaraki str, 3rd floor, 16675, Athens, Greece

Prof Eleni Zamba Papanicolaou, The Cyprus Institute of Neurology & Genetics, Nicosia, Cyprus

Dr Efthymios Dardiotis, UNIVERSITY HOSPITAL OF LARISSA, NEUROLOGY Department, Greece

Prof Shazia Maqbool, Department of Developmental and Behavioral Pediatrics, CH&ICH, Lahore, Pakistan

Prof Shahnaz Ibrahim, Department of Pediatrics and child health, Aga Khan University, Karachi, Pakistan

Prof Salman Kirmani, Department of Paediatrics & Child Health, The Aga Khan University, Karachi , Pakistan

Dr. Nuzhat Noureen Rana, Department of Paediatric Neurology, Children Hospital Complex and ICH, Multan, Pakistan

Dr. Osama Atawneh, Hilal Pediatric Hospital Hebron, Hebron West Bank, Palestine

Prof George Koutsis, Neurogenetics Unit, Neurology Department, Eginition Hospital, National and Kapodistrian University, Athens, Greece

Prof Salvatore Mangano, Unità di Neuropsichiatria Infantile, AOUP "P.Giaccone" Palermo, Italy

Dr Carmela Scuderi, Associazione Oasi Maria SS, 94018 Troina, Italy

Dr Eugenia Borgione, Associazione Oasi Maria SS, 94018 Troina, Italy

Dr Giovanna Morello, Institute of Neurological Sciences, National Research Council, Mangone, Italy

Dr Tanya Stojkovic, Institute of Myology, Hôpital La Pitié Salpêtrière, Paris, France

Prof Massimo Zollo, CEINGE, Biotecnologie Avanzate S.c.a.rl., Naples, Italy

Dr Gali Heimer, University Hospital of Tel Aviv, Tel Aviv, Israel

Prof Yves A. Dauvilliers, University Hospital Montpellier, Montpellier, France

Prof Pasquale Striano, Institute “Giannina Gaslini”, Genova, Italy

Dr Issam Al-Khawaja, Albashir University Hospital, Amman, Jordan

Dr Fuad Al-Mutairi, King Saud University, Riyadh, Saudi Arabia

Prof Sherifa Ahmed Hamed, Assiut University Hospital, Assiut, Egypt

Prof. Mohamed A. Abd El Hamed, Department of Neurology and Psychiatry, Assuit University Hospital, Assiut, Egypt.

Dr. Samson Khachatryan, "Somnus" Neurology Clinic Sleep and Movement Disorders Center, Yerevan, Armenia

Dr. Ulviyya Guliyeva, Mediclub clinic, Baku, Azerbaijan

Dr. Sughra Guliyeva, Mediclub clinic, Baku, Azerbaijan

Dr. Kamran Salayev, Azerbaijan State Medical University, Baku, Azerbaijan

Dr. Georgia Xiromerisiou, Department of Neurology, Medical School, University of Thessaly, Larissa, Greece

Dr. Liana Fidani, Department of Biology, Medical School, Aristotle University, Thessaloniki, Greece

Dr. Cleanthe Spanaki, Department of Neurology, Medical School, University of Crete, Heraklion, Greece

Prof. Mhammed Aguennouz, Department of Clinical and Experimental Medicine, University of Messina, Messina 98123, Italy

Prof. Gabriella Silvestri, Institute of Neurology, Università Cattolica del Sacro Cuore, Rome, Italy

Dr. Chingiz Shashkin, Kazakh National State University, Almaty, Kazakhstan

Dr. Nazira Zharkynbekova, Shymkent Medical Academy, Kazakhstan

Dr. Kairgali Koneyev, Kazakh National State University, Almaty, Kazakhstan

Prof. Abdullah Al-Ajmi, Neurology Unit, Department of Medicine, Al-Jahra Hospital, Kuwait

Prof. Shen-Yang Lim, Department of Biomedical Science, Faculty of Medicine, University of Malaya, Malaysia

Dr. Farooq Shaikh, Jeffrey Cheah School of Medicine and Health Sciences, Monash University Malaysia

Prof. Mohamed El Khorassani, Children's Hospital of Rabat, University of Rabat, Rabat 6527, Morocco

Prof. Arn M J M van den Maagdenberg, Leiden University Medical Center, Albinusdreef 2, Leiden 2333, Netherlands

Prof. Njideka U. Okubadejo, College of Medicine, University of Lagos (CMUL) & Lagos University Teaching Hospital, Idi Araba, Lagos State, Nigeria

Dr. Oluwadamilola O. Ojo, College of Medicine, University of Lagos, (CMUL) & Lagos University Teaching Hospital (LUTH), Idi Araba, Lagos State, Nigeria.

Prof. Kolawole Wahab, University of Ilorin Teaching Hospital (UITH), Ilorin, Kwara State, Nigeria.

Dr. Abiodun H. Bello, University of Ilorin Teaching Hospital (UITH), Ilorin, Kwara State, Nigeria.

Prof. Sanni Abubakar, Ahmadu Bello University, Zaria, Kaduna State, Nigeria.

Dr. Yahaya Obiabo, Delta State University Teaching Hospital, Oghara, Delta State, Nigeria.

Dr. Ernest Nwazor, Federal Medical Centre, Owerri, Imo State, Nigeria.

Dr. Oluchi Ekenze, University of Nigeria Teaching Hospital, Ituku-Ozalla, Enugu State, Nigeria.

Dr. Uduak Williams, University of Calabar Teaching Hospital, Calabar, Cross Rivers State, Nigeria.

Dr. Alagoma Iyagba, University of Port Harcourt Teaching Hospital, Port Harcourt, Rivers State, Nigeria.

Dr. Lolade Taiwo, Babcock University, Ilishan, Remo & Federal Medical Centre, Abeokuta, Ogun State, Nigeria.

Prof. Morenikeji Komolafe, Obafemi Awolowo University Teaching Hospital (OAUTH), Ile-Ife, Osun State, Nigeria.

Dr. Olapeju Oguntunde, Lagos University Teaching Hospital (LUTH), Nigeria.

Dr. Konstantin Senkevich, Almazov Medical Research Centre and Pavlov First Saint Petersburg State Medical University, Saint-Petersburg, Russia

Prof. Fowzan S Alkuraya, King Faisal Specialist Hospital and Research Center, Riyadh, Saudi Arabia

Dr. Ganieva Manizha, Avicenna Tajik State Medical University, Dushanbe, Tajikistan

Dr. Maksud Isrofilov, Avicenna Tajik State Medical University, Dushanbe, Tajikistan

Dr. Erin Torti, GeneDX, Gaithersburg, Maryland, USA.

Dr. Hoda Tomoum, Ain Shams Pediatrics Hospital, Ain-Shams University, Cairo, 11665, Egypt.

Dr. Amira Nabil, Human Genetics, Medical Research Institute, Alexandria University, Egypt.

Dr. Paola Nicolaides, American Medical Center, Nicosia, Cyprus.
